# Potential Inherent Stimulation of the Innate Immune System by Nucleic Acid Aptamers and Possible Corrective Approaches

**DOI:** 10.3390/ph11030062

**Published:** 2018-06-23

**Authors:** John G. Bruno

**Affiliations:** Operational Technologies Corporation, 4100 NW Loop 410, Suite 100, San Antonio, TX 78229, USA; brunobiotech@gmail.com; Tel.: +1-210-731-0015; Fax: +1-210-731-0041

**Keywords:** aptamer, CpG, innate immunity, methylation, oligonucleotide, Toll-like receptors

## Abstract

It is well known that unmethylated 2′-deoxycytidine-phosphate-2′-guanine (CpG) sequences alone or in longer DNA and RNA oligonucleotides can act like pathogen-associated molecular patterns (PAMPs) and trigger the innate immune response leading to deleterious cytokine production via Toll-like receptors (TLRs). Clearly, such CpG or CpG-containing sequences in aptamers intended for therapy could present very damaging side effects to patients. Previous antisense oligonucleotide developers were faced with the same basic CpG dilemma and devised not only avoidance, but other effective strategies from which current aptamer developers can learn to ameliorate or eliminate damaging CpG effects. These strategies include obvious methylation of cytosines in the aptamer structure, as long as it does not affect aptamer binding in vivo, truncation of the aptamer to its essential binding site, backbone modifications, co-administration of antagonistic or suppressive oligonucleotides, or other novel drugs under development to lessen the toxic CpG effect on innate immunity.

## 1. Introduction and Background

**Figure 1:**
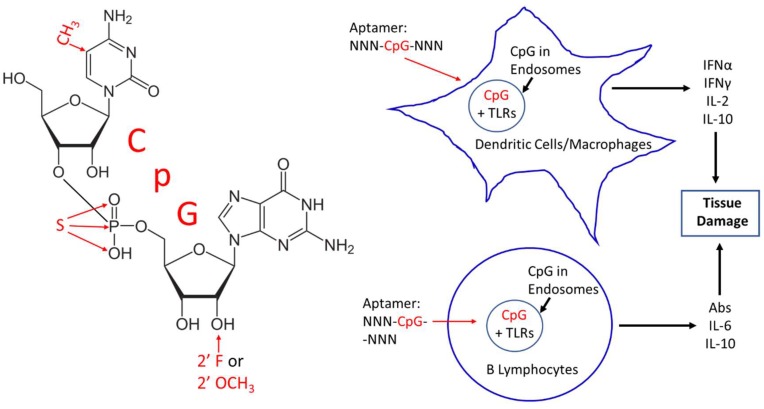
**Left**—2013 molecular structure of 2′-deoxycytidine-phosphate-2′-guanine (CpG) showing several potential remedies for CpG toxicity in red text such as methylation at the 5 position of cytosine, phosphorothioate (S or sulfur for oxygen) substitution in the phosphate linkage and 2′ fluoro (F) or 2′ O-methyl (O-CH_3_) modifications. **Right**—schematic illustration of the possible in vivo toxicity mechanism caused by CpG segments in aptamers upon entry to endosomes where CpG segments can bind the Toll-like receptors (TLRs) 3, 7, 8, and 9 to induce cytokine (e.g., interferons (IFN) alpha and gamma, interleukins (IL) 2, 6, and 10) production and antibody secretion, potentially leading to unintended tissue damage.

By virtue of being composed of DNA or RNA, aptamers have basically been considered nonimmunogenic in the sense that they generally do not induce strong antibody production [1]. While quite complex and not yet totally understood, it has long been known that other potentially therapeutic oligodeoxynucleotides (ODN) including antisense ODNs containing the unmethylated dinucleotide sequence 2′-deoxycytidine-phosphate-2′-guanine (CpG), as shown in Figure 1, could stimulate the innate immune system via several of the Toll-like receptors (TLRs) including TLR3, 7, 8, and 9 in endosomes, because unmethylated CpG suggests the presence of invading bacterial or viral nucleic acids [2,3,4,5,6,7]. In effect then, CpG sequences alone or in the midst of other longer sequences represent potential pathogen-associated molecular patterns (PAMPs) similar to lipopolysaccharides or other bacterial- and viral-associated molecules. The endocytosis of CpG or other oligonucleotides containing CpG in B lymphocytes as well as monocytes, macrophages, dendritic, and other immune cells can trigger several key cytokines [2,3,4,5,6,7], which may be deleterious (Figure 1) and, therefore, counterproductive to the intent of the therapeutic aptamer or antisense ODN. CpG immunostimulation can be so strong that CpG alone and in other ODNs have been investigated as possible vaccine adjuvants with promising results [7,8,9].

To be clear, the use of the abbreviation CpG throughout this review will generally indicate the 2′-deoxyribose or DNA version of the nucleoside tandem, although the 2′-oxy or ribose-containing RNA version of CpG (Figure 1) can also stimulate the innate immune system [6]. To make matters more complex and bewildering, the context of surrounding nucleotides in which CpG motifs reside is important for determining the level of innate immune system stimulation, with G-rich or GC-rich regions being far less stimulatory for the innate immune system [2,5,7]. Various host species (e.g., murine, human, etc.) are also differently affected by different CpG-containing sequences [10] as one might expect due to their genetic differences. 

One might reasonably wonder as well if orientation of the CpG motif can impact its ability to stimulate host innate immune systems. The answer appears to be that, yes, orientation is important because 5′-CpG-3′ stimulates cytokine production, but the reverse (5′-GpC-3′) by itself or in the context of other nucleotides does not appear to activate innate immunity [10,11]. While the rich, but often bewildering, CpG immune response literature is fascinating on its own, it is not the subject of this brief review. Rather, for the aptamer developer, avoiding CpG sequences or developing countermeasures much as antisense developers as has been previously done [12,13,14], is the subject of this review.

While much of this review is focused on how to avoid or lessen the toxic effects of CpG sequences in aptamers, one should first empirically determine if there is actually innate immune system activation by a particular aptamer before undertaking the more complicated molecular engineering approaches outlined herein. Outside of animal testing models, to aid in empirical testing of aptamer TLR or other innate immune system activation, several in vitro systems have been developed and published. Avci-Adali et al. [15,16] have developed in vitro systems to assess cytokine production levels in response to particular aptamers. Other researchers have utilized green fluorescent protein (GFP)-linked reporter systems in macrophages [4,5] and other immune cells to indicate TLR activation by ODNs such as aptamers.

## 2. CpG Toxicity Based on Route of Administration and Molecular Context 

The only FDA-approved aptamer on the market is Macugen^®^ (pegaptanib) for treatment of age-related macular degeneration. Macugen is administered by intraocular (vitreous humor) injection, which may in part account for the minimal cytokine production and negligible side effects of Macugen. Other injection sites beside the eye or other routes of administration (i.e., intramuscular or intravenous, etc.) might be expected to encounter more immune cells and lead to a proportionately greater innate immune response. Interestingly, Macugen contains two CpG sequences [17]. However, Macugen’s two CpG sequences are hydrogen bonded to one another in a very stable double-stranded stem region of the aptamer according to computer-generated stem-loop models of Macugen [17], thereby possibly neutralizing their ability to bind TLRs or trigger innate immunity. In addition, Macugen contains 2′-O-methyl and 2′-fluoro modifications [17] (Figure 1) shown to confer nuclease resistance and ameliorate TLR activation [14,17,18]. 

**Figure 2:**
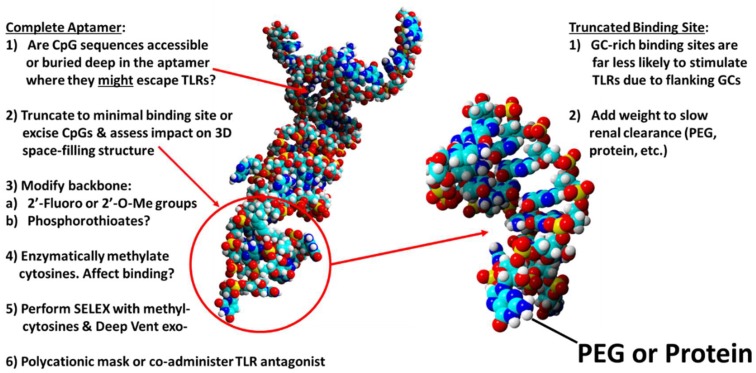
Three-dimensional (3-D) space-filling models of one of the author’s developed aptamers using YASARA [21] to analyze the accessibility of potentially inflammatory CpG sequences and alterations to the 3-D structure of the putative binding site (**left**) once it is excised from the complete aptamer (**right**). The figure also summarizes a list of approaches to evaluating and rectifying potential CpG toxicity problems. PEG = polyethylene glycol.

While 2-dimensional aptamer models can be useful, in the study of CpG accessibility to TLRs, 3-dimensional (3-D) topology models are even more useful (e.g., Figure 1) for determining if a CpG region is theoretically accessible or resides in an invaginated pocket (tucked up inside) of an aptamer tertiary structure. Accurate protein or other macromolecular 3-D structure prediction is complex and ranks among the most difficult problems in mathematics [19] whether analyzed in vacuo or in the more realistic hydrated state with physiologic ion concentrations. In addition, most of the 3-D computer programs for macromolecular folding are designed for proteins. Thus, in the past, accurate 3-D aptamer modeling and docking analyses were expensive tasks [20]. However, if one is willing to accept a little less accurate model for rough determination of CpG accessibility, then less expensive and even free web-based software such as YASARA can be used or linked together to produce 3-D surface models such as those depicted in Figure 2 [21,22,23,24]. Figure 2 also illustrates how a second generation truncated derivative aptamer consisting of a minimum essential binding site that retains its original tertiary shape might be synthesized to eliminate one or more toxic CpG segments. Of course, the lighter second generation derivative aptamer (binding site) may require increased weight in the form of polyethylene glycol (PEG) or protein attachment to aid in slowing kidney clearance and enhancing aptamer half-life in vivo [1].

On a simpler level, molecular context can also imply the effects of bases flanking a CpG sequence. As aforementioned, a CpG segment in G-rich or GC-rich regions of an ODN are not inflammatory or not nearly as inflammatory as in other regions [2,5,7], and GC-rich regions are characteristic of aptamer binding sites because they lend 3-D stability to the binding pockets. Particular examples of short ODNs (hexamers to octamers especially) containing CpG segments and their level of innate immune system activation are described in many places in the literature [2,5,7]. Again, it probably behooves the aptamer developer to test a given aptamer’s level of innate immune system activation empirically [5,15,16].

## 3. Corrective Strategies

Assuming that an aptamer developer cannot excise a toxic CpG-containing segment from a candidate aptamer, other corrective strategies can be attempted. Perhaps, the most obvious and effective strategy is to methylate the culpable cytosine in CpG (Figure 1) with CpG methylase or methyltransferase [5] as long as this does not affect aptamer binding affinity or specificity for its cognate target. To avoid this potential post-SELEX modification binding problem, one could consider SELEX with 5-methylcytosine incorporation originally to guarantee target binding and proper methylation. This would require a more permissive or promiscuous form of Taq polymerase such as Deep Vent^®^ exo-DNA polymerase [25] to incorporate 5-methylcytosines into the aptamer. Deep Vent exo- has been shown to incorporate even fluorophore-labeled nucleotides into aptamers or other ODNs [26,27], thus, incorporation of methylated cytosines should not be problematic during SELEX aptamer development.

If methylation does not completely eliminate a CpG toxicity issue, aptamer developers can also modify aptamer backbones since 2′-*O*-methyl groups [14] and 2′-fluoro [18] groups on the sugar moieties of nucleic acids have been shown to lessen the innate immune response. Although more controversial [2], phosphorothioate backbones may also decrease the innate response of CpG sequences [28]. Another approach is to add a second material to bind or mask the CpG segments of an aptamer. Sullenger’s laboratory at Duke University Medical Center experimented with various polycationic materials such as poly-l-lysine, third generation (G3) polyamidoamine (PAMAM) dendrimer derivatives, and other such compounds as general charge-based electrostatic binding agents to bind and mask the polyanionic phosphate backbone of nucleic acids in circulation [29]. Of course, this rather nonspecific masking approach assumes that the aptamer will have a greater affinity for its cognate target than the polycationic masking agent, thereby allowing the aptamer to dissociate from the masking agent and associate with its cognate target, which is most often thermodynamically favorable and, therefore, theoretically a rather safe bet in most cases. 

Another class of somewhat more specific competitive agents for co-administration with a potentially inflammatory aptamer is that of “suppressive” oligonucleotides. As previously discussed, there are G-rich or GC-rich suppressive ODNs [30,31], RNA oligonucleotides [32], or other TLR-suppressive drugs in the development pipeline [33,34,35] to antagonize TLRs and ameliorate their deleterious effects. Again, aptamers are often G- or GC-rich in their stabilized binding sites, so that if a CpG segment exists in the binding pocket, it may be cancelled out by TLR-suppressive G- or GC-rich regions in proximity to the CpG locus.

## 4. Conclusions 

Although some critics continue to question the future of aptamers, especially as pharmaceuticals, the future of aptamers still appears bright due to the advantages of aptamers over antibodies such as obviating host animals during development and production to reduce overall costs and greater batch to batch reproducibility and facile post-production modifications to “fine tune” performance [36]. There are so many promising applications for aptamers in the areas of enhanced drug delivery [37], therapy of antibiotic-resistant bacteria [38,39,40], deadly viruses [41,42,43], and cancers [44,45], inhibition of venoms [46] and biotoxins [47], regulation of blood clotting [48], drug transport across the blood-brain barrier [49], and stem cell differentiation or transdifferentiation induction [36,49], just to name a few potential uses. With so much promise in so many areas of critical medical need, the aptamer community cannot let CpG toxicity inhibit aptamer development progress. Of course, even the most innocuous portions of therapeutic conjugates such as PEG can lead to adverse reactions and even death due to pre-existing or induced antibodies or other immune mechanisms in a very small percentage of patients [50]. However, where there is a will, there is a way to overcome such problems, including substitution of PEG adjuncts with common blood proteins such as serum albumins to protect the 3′ end and add weight [37,38,39], thereby slowing kidney clearance (Figure 1). By analogy, and hopefully, this mini-review will raise awareness of potential CpG toxicity and provide some pragmatic approaches to avoiding or ameliorating potential activation of the innate TLR-pathways that lead to undesired inflammatory responses, thus giving aptamers a better chance for future United States FDA and other worldwide medical approvals.
